# Assessment of diagnostic and therapeutic vitrectomy for vitreous opacity associated with uveitis with various etiologies

**DOI:** 10.1097/MD.0000000000009491

**Published:** 2018-01-12

**Authors:** Tomohito Sato, Rina Kinoshita, Manzo Taguchi, Sunao Sugita, Toshikatsu Kaburaki, Yutaka Sakurai, Masaru Takeuchi

**Affiliations:** a Ophthalmology, National Defense Medical College, Tokorozawa, Saitama; b Laboratory for Retinal Regeneration, RIKEN Center for Developmental Biology, Kobe, Hyogo; c Department of Ophthalmology, The University of Tokyo Hospital, Tokyo, Japan.

**Keywords:** diagnostic vitrectomy, infectious uveitis, therapeutic vitrectomy, uveitis, vitrectomy, vitreous opacity

## Abstract

Vitreous opacity (VO) is a common feature of intermediate uveitis, posterior uveitis, and panuveitis. Fundus observation is critical for determining the etiology of uveitis, however, is often interfered with VO. In these clinical settings, vitrectomy contributes to a correct diagnosis and guides alternative management strategies. The purpose of this study was to evaluate the diagnostic yield and surgical outcome of vitrectomy in uveitic patients with VO and compare the visual outcome between infectious and noninfectious uveitis. Forty-five eyes with uveitis-associated VO underwent diagnostic and therapeutic vitrectomy, and etiological diagnosis of uveitis was confirmed in 34 of 45 eyes (75.6%). The diagnoses were infectious uveitis in 13 eyes (28.9%), noninfectious uveitis in 21 eyes (46.7%), and unidentified uveitis in 11 eyes (24.4%). Visual acuity (VA) improvement rates at 6 months after surgery were 69.2%, 76.2%, and 90.9% in the infectious, noninfectious, and unidentified uveitis groups, with no significant difference among 3 groups. Significant decrease in inflammation score after vitrectomy was observed only in the infectious uveitis group. This study demonstrated that diagnostic vitrectomy for inflammatory eyes with VO of unknown etiology was effective in infectious and noninfectious uveitis, and the therapeutic effect of VA improvement was observed in both types of uveitis.

## 1 Introduction

Determining the etiology of uveitis is critical because of the different therapeutic and prognostic implications for individual disease entities. The diagnosis of etiology is based on a combination of history, clinical examinations, and laboratory and radiological findings. However, difficulties in diagnosis arise in cases with an atypical history, atypical clinical presentation, and complications impeding conclusive diagnostic workup. Difficulties in diagnosis have implications from a therapeutic standpoint. Vitreous opacity (VO) is a common feature of intermediate uveitis, posterior uveitis, and panuveitis. VO interferes with fundus observation especially in the acute phase, and occurs in both infections and noninfectious uveitis.^[1]^ Even though the presence of VO hampers a definitive diagnosis, it may be necessary to treat with local and systemic immunosuppression according to the presumptive diagnosis of uveitis. In some cases, the diagnosis is further confused by persistence or aggravation of intraocular inflammation, which may raise concern for an infectious or neoplastic etiology. In these clinical settings, vitrectomy allows detailed fundus observation and analysis of vitreous samples. In addition to providing therapeutic options such as clearance of inflammatory factors accumulated in the vitreous and restoration of transparency,^[2–5]^ vitrectomy contributes to a correct diagnosis and guides alternative management strategies. In 1979, Diamond and Kaplan^[6]^ reported the therapeutic effects of vitrectomy on uveitis by removing the vitreous gel containing inflammatory factors. In 1981, Carroll and Franklin^[7]^ advocated the usefulness of diagnostic vitrectomy for uveitis of unknown etiology. Advances in surgical and laboratory techniques in the past decades have expanded the indications of diagnostic and therapeutic vitrectomy. The principal methods of diagnostic vitrectomy at the early phase of disease were cytological and culture analyses using vitreous specimens. Recently, polymerase chain reaction (PCR), which is effective to detect pathogenic organisms using small amounts of vitreous samples, is widely used together with cytological and culture analyses in diagnostic vitrectomy. The development of multiplex PCR further allows comprehensive examinations of humoral factors with the same quantity of sample used in conventional PCR.^[8]^ From the technical viewpoint, the availability of new microincision sutureless vitrectomy technology, wide-angle microscope viewing systems, and pharmacological agents has reduced operative invasiveness, shortened surgical time, and improved surgical outcome.^[9–11]^ These achievements promote the use of therapeutic vitrectomy in patients with VO caused by uveitis of unknown etiology.^[12–15]^ Therefore, the diagnostic yield and therapeutic effects of vitrectomy in uveitic eyes with VO are improving, although surgical outcome in cases of infectious uveitis, especially endophthalmitis, remains unfavorable.

The purpose of this study was to evaluate the diagnostic yield and surgical outcome of vitrectomy in uveitic patients with VO and compare the visual outcome between infectious and noninfectious uveitis.

## 2 Materials and methods

### 2.1 Subjects

**Figure 1:**
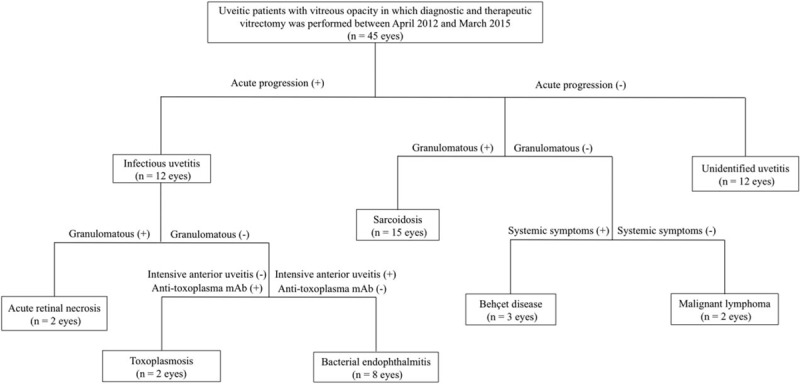
A flowchart of presumptive diagnosis responsible for vitreous opacity.

This retrospective observational study was approved by the institutional review board of National Defense Medical College, Saitama, Japan. We retrospectively reviewed the clinical records of 45 eyes (31 patients) with uveitis in which diagnostic and therapeutic vitrectomy was performed for 45 eyes with VO between April 2012 and March 2015 in National Defense Medical College. The age (mean ± SD) was 66.9 ± 12.0 years (range, 40–83), and gender (male/female) ratio was 13/32. All cases were followed for >6 months after vitrectomy. Figure 1 depicts a flowchart of presumptive diagnosis responsible for VO. A total of 45 eyes were first classified with or without active progression resistant for conventional anti-inflammatory agents. Twelve eyes (26.7%) with active progression were suspected as infectious uveitis, in which 2 eyes (4.4%) of granulomatous uveitis were diagnosed as acute retinal necrosis (ARN), 2 eyes (4.4%) of nongranulomatous uveitis with anti-toxoplasma gondii mAb were as ocular toxoplasmosis, and 8 eyes (17.8%) of nongranulomatous uveitis without anti-toxoplasma gondii mAb in addition to intensive cell infiltration into the anterior chamber were as bacterial endophthalmitis. Eyes without active progression were classified into granulomatous and nongranulomatous uveitis, or unidentified uveitis, in which 15 eyes (33.3%) of granulomatous uveitis were diagnosed as ocular sarcoidosis, 3 eyes (6.7%) of nongranulomatous uveitis with systemic symptoms, such as oral ulcer, skin lesions, and genital ulcer, were as behçet disease, and 2 eyes (4.4%) of nongranulomatous uveitis without systemic symptoms were malignant lymphoma.

### 2.2 Surgical techniques

Standard 3-port 25-gauge pars plana vitrectomy (Constellation Vitrectomy System; Alcon Laboratories, Fort Worth, TX) was performed. For 32 eyes, phacoemulsification with a 2.2-mm sutureless transconjunctival single-plane sclerocorneal incision was combined. Foldable acrylic lens, SN60WF (Alcon, Inc.) or NY-60 (HOYA Inc., Tokyo, Japan), were implanted in the bag with injectors before subsequent vitrectomy. The microcannulaes were inserted through the conjunctiva into the eye (3.5 mm posterior to the limbus), and vitrectomy was performed using a wide viewing system (BIOM, Oculus, Wetzlar, Germany). Meniscus contact lens (Hoya, Tokyo, Japan) was used only for macular surgery. Brilliant blue G-assisted inner limiting membrane peeling was performed in all eyes with epiretinal membrane with or without cystoid macular edema. Scleral depression for shaving the vitreous base, endolaser photocoagulation, air exchange, and scleral buckling were performed as necessary. At the end of the vitrectomy, 4.0 mg of triamcinolone acetonide was injected intraocularly except eyes presumed as infectious uveitis. For 8 eyes with presumptive diagnosis of endophthalmitis, intravitreal injection of vancomycin 1 mg/0.1 cm^3^ and ceftazidime 2.25 mg/0.1 cm^3^ were performed before and after surgery, and vancomycin (20 μg) and ceftazidime (40 μg) were added to infusion fluid of balanced salt solution (BSS; Santen, Osaka, Japan) during vitrectomy in addition to systemic administration of ceftazidime. Although 5 eyes out of the 8 eyes were pseudophakic eyes suspected as postoperative endophthalmitis, inner capsule was irrigated by excising the posterior capsule in circle, and intraocular lens were not extracted. In 2 eyes with presumptive diagnosis of acute retinal necrosis, Acyclovir (1500–2250 mg/d) and prednisolone (30 mg/d) were systemically administrated, and lensectomy, silicone oil tamponade, and encircling scleral buckle were performed in combination with vitrectomy.

Patients usually had follow-up evaluations at day 7 after surgery and every month thereafter. At each follow-up, complete ophthalmic examination was performed, with additional fluorescein angiography and optical coherence tomography when required.

### 2.3 Medications

Medications administrated before vitrectomy were continued during the surgical period. For eyes suspected bacterial endophthalmitis, intravitreal injection of the peptide antibiotic vancomycin (1.0 mg/0.1 mL) in combination with the β-lactam antibiotic ceftazidime (2.25 mg/0.1 mL) and broad-spectrum antimicrobial therapy were performed before subsequently emergent vitrectomy.^[16]^ After surgery, systemic antimicrobial agents susceptive for the causative pathogen were also provided for several days to ensure eradication of the infection. In other cases, preoperative systemic medication was continued after surgery, and eye drops of 0.1% betamethasone sodium phosphate and 0.5% moxifloxacin ophthalmic solution were provided 4 times per day for 2 months. Bromfenac sodium hydrate was also used for eyes with cataract surgery. Thereafter, eye drops used before surgery were restarted. Systemic medications were initiated or increased according to the grade of ocular inflammation after surgery, and was discontinued when postoperative inflammation was resolved.

### 2.4 Sample Collection

Approximately, 0.2 to 0.5 mL of undiluted vitreous fluid was obtained using a 25 G vitreous cutter inserted into the mid-vitreous cavity at the beginning of vitrectomy before active infusion. Samples were centrifuged to remove cellular components. Each sample was aliquoted into 4 sterile tubes and stored at −70 °C until analysis. No complications associated with vitreous sampling were observed.

### 2.5 Diagnosis

All vitreous specimens were used for microscopic examination, bacterial and fungal cultures, and the following examinations. Regarding comprehensive PCR analysis, genomic DNA extracted from the vitreous sample was analyzed first by multiplex PCR and quantitative real-time PCR for human herpes viruses (HHVs) 1 to 8 and toxoplasma.^[8]^ Subsequently, samples were examined by broad-range real-time PCR for bacterial 16S and fungal 18S/28S ribosomal DNA (rDNA). Extracted DNA was also examined for amplification of the immunoglobulin heavy chain (IgH) gene to detect IgH gene rearrangement. Moreover, interleukin (IL)-6 and IL-10 levels in the vitreous samples were measured by immunosorbent assay. A diagnosis of malignant lymphoma was based on IL-10/IL-6 ratio >1.0 and/or detection of IgH gene rearrangement.^[17]^

### 2.6 Outcomes and data analysis

Descriptive statistics included mean, standard deviation, median, and range. The decimal best corrected visual acuity measured using Snellen chart was converted into logarithm of minimum angle of resolution (logMAR) for statistical analysis. Ocular inflammation based on the degrees of cells and flare in the anterior chamber was scored on a scale of 0 to 4 as reported by the Standardization of Uveitis Nomenclature (SUN) Working Group.^[1]^ Patients were classified into 3 groups based on the diagnosis after vitrectomy; infectious uveitis, noninfectious uveitis, and unidentified uveitis, for data analysis. LogMAR and ocular inflammation scores before and at 1, 3, or 6 months after surgery were compared by Wilcoxon signed-rank test. Comparison among 3 groups was analyzed by one-way analysis of variance (ANOVA) and Steel-Dwass multiple comparison test. All analyses were performed by JMP version 12, and *P* < .05 was considered to be statistically significant.

## 3 Results

### 3.1 Positive predictive values of infectious and noninfectious uveitis in presumptive diagnosis before surgery

Positive predictive value (PPV) of infectious uveitis in presumptive diagnoses before surgery is shown in Table 1. Infectious uveitis in presumptive diagnoses was 15 eyes, in which 12 eyes were infectious uveitis in definitive diagnosis, and the PPV was 75%. PPV of ARN and ocular toxoplasmaosis was 100%, while that of bacterial endophthalmitis was 62.5%. Table 2 is PPV of noninfectious uveitis in presumptive diagnoses before surgery. Noninfectious uveitis in presumptive diagnoses was 20 eyes, in which 19 eyes were noninfectious uveitis in definitive diagnosis, and the PPV was 95%. PPV of malignant lymphoma and behçet disease was 100%, while that of sarcoidosis was 93.3%.

**Table 1:**
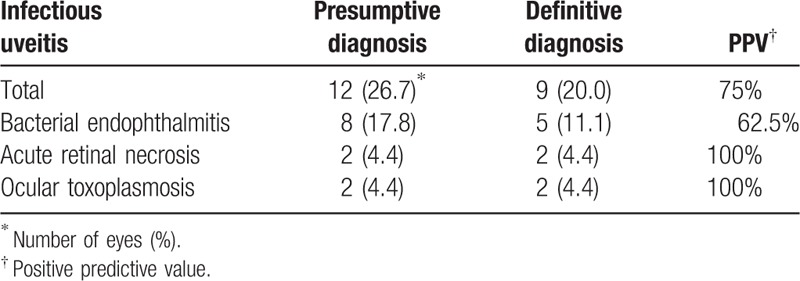
Positive predictive value (PPV) of infectious uveitis in presumptive diagnoses before surgery.

**Table 2:**
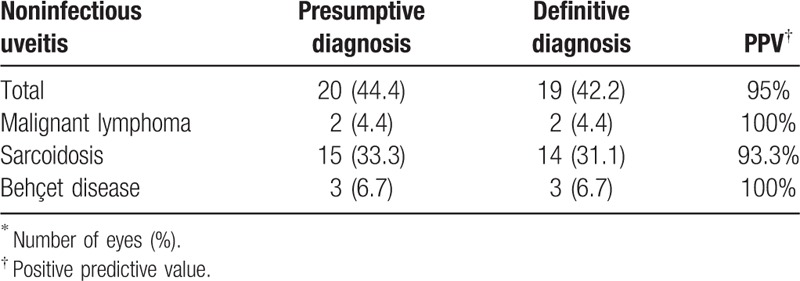
Positive predictive value (PPV) of noninfectious uveitis in presumptive diagnoses before surgery.

### 3.2 Diagnostic yield and etiology of unidentified uveitis before surgery

Table 3 shows definitive diagnosis and diagnostic yield of unidentified uveitis before surgery. In 13 eyes of unidentified uveitis in presumptive diagnosis before surgery, 7 eyes were still unidentified uveitis in definitive diagnosis. But, 6 eyes were diagnosed, and diagnostic yield was 46.2%. Among 6 eyes newly diagnosed, 3 eyes were diagnosed as cytomegalovirus (CMV) retinitis, 2 eyes were malignant lymphoma, and 1 eye was EB-associated uveitis.

**Table 3:**
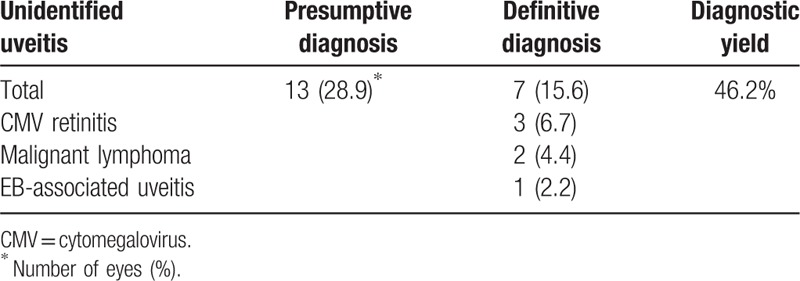
Definitive diagnosis and diagnostic yield of unidentified uveitis before surgery.

### 3.3 Characteristics of uveitic patients with vitreous opacity in definitive diagnosis

The characteristics of uveitic patients with VO classified into infectious, noninfectious, and unidentified uveitis groups by definitive diagnosis after surgery are shown in Table 4. The noninfectious uveitis group was significantly younger than the infectious or unidentified uveitis group. There was almost the same number of male and female patients in the infectious uveitis group, while there were more female than male patients in the noninfectious and unidentified uveitis groups, although there was no significant difference. In addition, logMAR and inflammation scores in infectious uveitis group were significantly higher than those of noninfectious uveitis group.

**Table 4:**
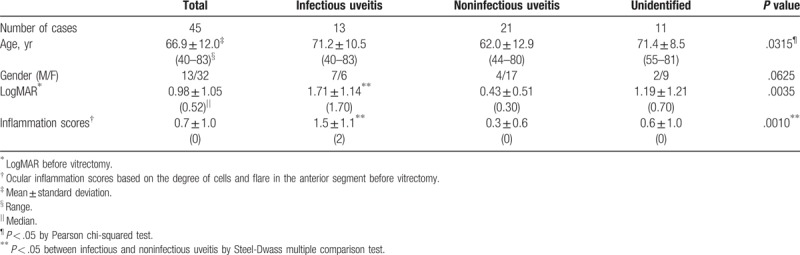
Characteristics of uveitic patients with vitreous opacity in definitive diagnosis after surgery.

### 3.4 Effects of vitrectomy on logMAR and inflammation scores in infectious, noninfectious, and unidentified uveitis

**Figure 2:**
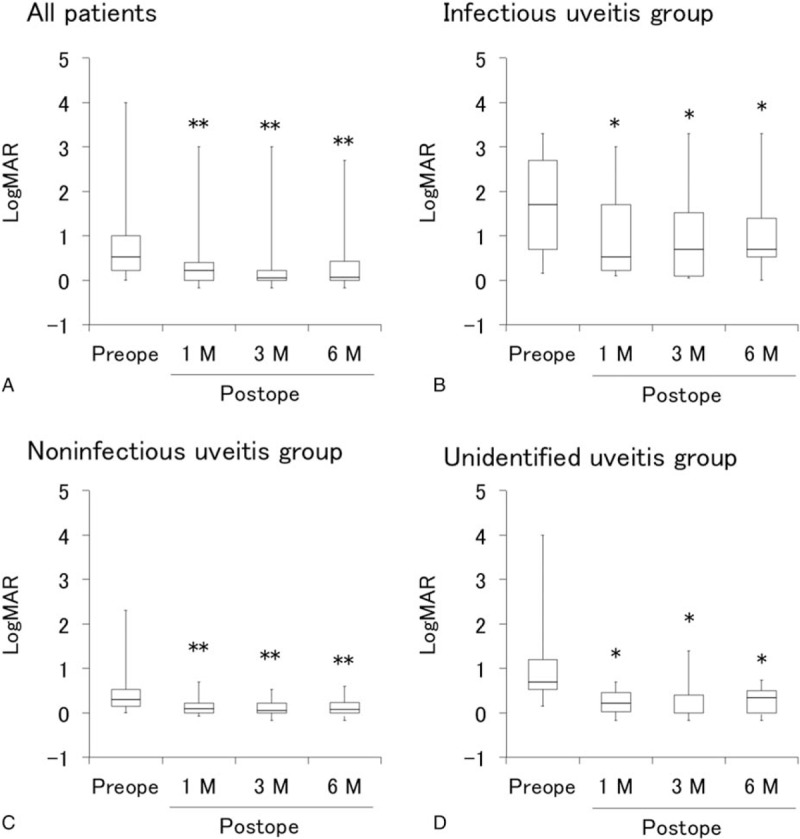
Visual acuity before and after vitrectomy in uveitis with different etiologies. Box plots of logMAR before vitrectomy and at 1, 3, and 6 months after vitrectomy in all patients (A), infectious uveitis group (B), noninfectious uveitis group (C), and unidentified uveitis group (D). ∗*P* < .05, ∗∗*P* < .005 by Wilcoxon signed-rank test. LogMAR = logarithm of minimum angle of resolution.

**Figure 3:**
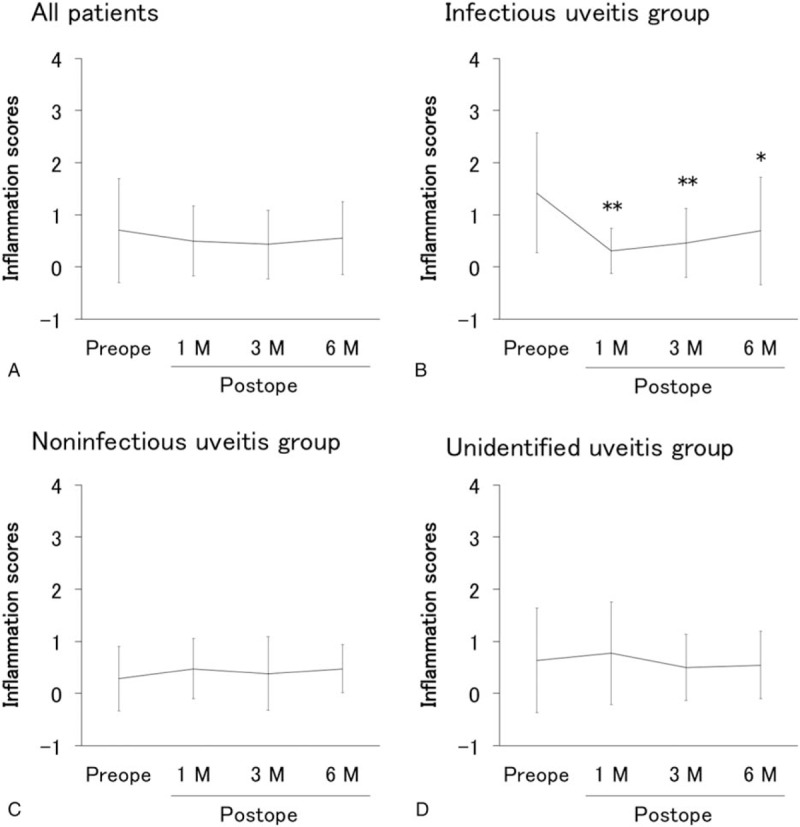
Inflammation scores before and after vitrectomy in uveitis with different etiologies. Inflammation scores (mean ± standard deviation) before vitrectomy and at 1, 3, and 6 months after vitrectomy in all patients (A), infectious uveitis group (B), noninfectious uveitis group (C), and unidentified uveitis group (D). ∗*P* < .05, ∗∗*P* < .005 by Wilcoxon signed-rank test.

LogMAR in all patients and in each infectious, noninfectious, unidentified uveitis group before and at 1, 3, and 6 months after vitrectomy are presented in Fig. 2, and the corresponding data for inflammation score are shown in Fig. 3. Visual acuity (VA) improved significantly from 1 month after vitrectomy compared with before vitrectomy, and remained stable until 6 months in all groups, and in each infectious, noninfectious, and unidentified uveitis group (Fig. 2). On the other hand, although inflammation scores after vitrectomy did not changed in noninfectious or unidentified uveitis group compared with those before surgery, a significant decrease in inflammation score was observed in the infectious uveitis group from 1 month after vitrectomy compared with before vitrectomy (Fig. 3).

### 3.5 Comparison of logMAR and inflammation scores before and after vitrectomy between infectious, noninfectious, and unidentified uveitis groups

**Figure 4:**
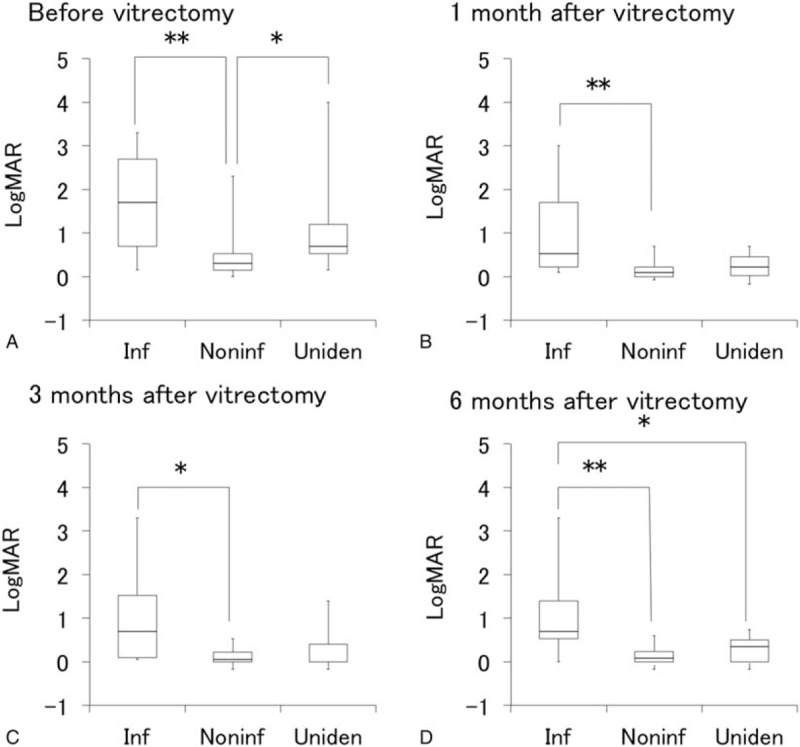
Comparison of visual acuity before and after vitrectomy among uveitis with different etiologies. Box plots of logMAR in infectious uveitis group (Inf), noninfectious uveitis group (Noninf), and unidentified uveitis group (Uniden) before vitrectomy (A) and at 1 month (B), 3 months (C), and 6 months (D) after vitrectomy. ∗*P* < .05, ∗∗*P* < .005 by Steel-Dwass multiple comparison test. LogMAR = logarithm of minimum angle of resolution.

**Figure 5:**
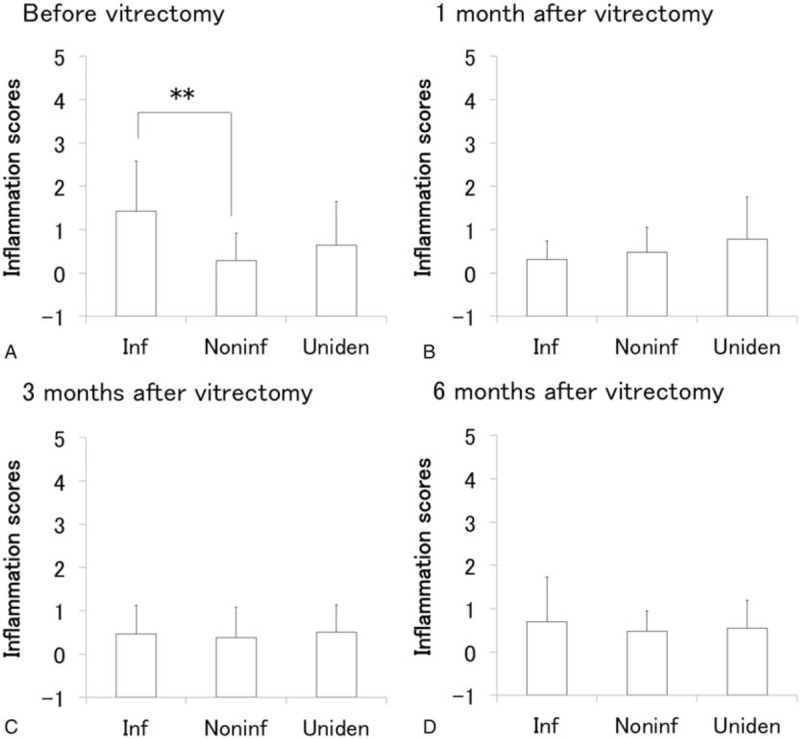
Comparison of inflammation scores before and after vitrectomy among uveitis with different etiologies. Inflammation scores (mean ± standard deviation) in infectious uveitis group (Inf), noninfectious uveitis group (Noninf), and unidentified uveitis group (Uniden) before vitrectomy (A) and at 1 month (B), 3 months (C), and 6 months (D) after vitrectomy. ∗*P* < .05, ∗∗*P* < .005 by Steel-Dwass multiple comparison test.

LogMAR before and after vitrectomy in the infectious, noninfectious, unidentified uveitis groups are presented in Fig. 4, and inflammation scores are shown in Fig. 5. VA was significantly lower in the infectious uveitis group compared with the noninfectious uveitis group before vitrectomy as well as at 1, 3, and 6 months after vitrectomy (Fig. 4). On the other hand, while pre-vitrectomy inflammation score in the anterior segment was significantly higher in the infectious uveitis group than in the noninfectious uveitis group, the post-vitrectomy scores were not significantly difference between the 2 groups (Fig. 5).

### 3.6 Improvement rate of visual acuity (VA) at 6 months after vitrectomy in infectious, noninfectious, and unidentified uveitis groups

**Figure 6:**
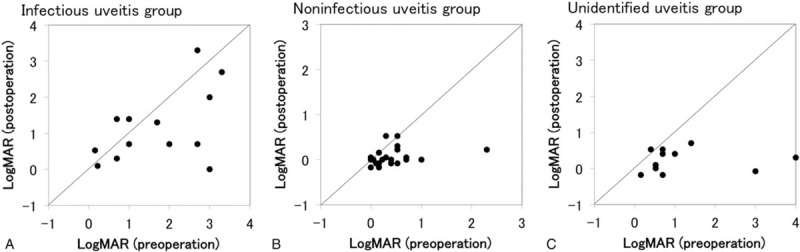
Comparison of pre- and post-vitrectomy visual acuity in uveitis with different etiologies. Plots of logMAR before vitrectomy (*x*-axis) versus logMAR at 6 months after vitrectomy (*y*-axis) in individual eyes of infections uveitis group (A), noninfectious uveitis group (B), and unidentified uveitis group (C). LogMAR = logarithm of minimum angle of resolution.

Plots of logMAR before vitrectomy versus logMAR at 6 months after vitrectomy in individual patients are shown in Fig. 6. The rate of visual acuity (VA) improvement at 6 months after surgery was 9 of 13 eyes (69.2%) in the infectious uveitis group, 16 of 21 eyes in the noninfectious uveitis group (76.2%), and 10 of 11 eyes (90.9%) in the unidentified uveitis group. VA improvement rate was the lowest in the infectious uveitis group, but there was no significant difference among 3 groups.

## 4 Discussion and conclusions

For a patient with VO in whom causative agent or etiology is not identified, differential diagnosis is important to distinguish the true etiology from various possible diseases. The diagnostic vitrectomy used in this study was useful to rule out various etiologies causing VO and consequently allows choices of appropriate therapeutic options.^[13,15,18]^

When bacterial endophthalmitis is suspected as a specific cause of VO, broad-spectrum antimicrobial therapy is initiated. The potential causative pathogens were detected 5 of 8 eyes (62.5%) in this study. If diagnostic vitrectomy detects a specific organism, therapy directed toward the offending organism can be initiated, thereby minimizing exposure of the patient to unnecessary medications and their potential adverse effects. On the other hand, since newer diagnostic tests using vitreous specimens were performed in all the patients with VO reviewed in this study, infectious uveitis and malignant lymphoma were excluded in 71.2% of the other cases, and the etiology was identified in 37.8% of them by removal of VO which enabled fundus examinations. Therefore, anti-inflammatory treatment was continued with greater confidence for these eyes.

Although many reports have examined diagnostic vitrectomy for uveitis, the diagnostic yields of these studies varied widely,^[3–5,7,19,20]^ due to differences in selection criteria of patients with uveitis, surgical techniques, and procedures or methods for analysis of vitreous specimens. In the present study, 14 eyes of sarcoidosis and 3 eyes of behçet disease were confirmed fundus findings by surgical removal of VO in addition to 17 eyes diagnosed by analysis of vitreous specimens, and the overall diagnostic yield of vitrectomy was 75.6%, and vitreous fluid analyses yielded positive results in 37.8% of the cases (28.9% was infectious uveitis and 8.9% was malignant lymphoma). These results were lower than those of previous reports, contrary to our expectation that newer diagnostic tests such as comprehensive PCR analysis, IL-6/IL-10 ratio and IgH using vitreous specimens performed in the present study would improve the diagnostic yield.^[17,21]^ Low cut rate of 600 cpm or less and low aspiration,^[22]^ and cell block specimens^[23,24]^ have been reported to raise the diagnostic positive ratio of vitreoretinal lymphoma. Although we have used cell block specimens for microscopic examination, cut rate was fixed at 7500 cpm and was not decreased to 600 cpm or less.

Other possible reason is that the patients with VO reviewed in this study had diverse etiologies and not limited to malignant lymphoma and endophthalmitis.

In addition, eyes presumptively diagnosed as bacterial endophthalmitis by the clinical course and ocular findings were excluded in definitive diagnosis, if the pathogen was not identified by microscopic examination, bacterial and fungal cultures, or broad-range real-time PCR, even though the intraocular findings during surgery were compatible with bacterial endophthalmitis and postoperative antibacterial agents were effective in their therapy. Therefore, PPV of infectious uveitis (75%) was lower than that of noninfectious uveitis (95%), which would be 1 factor of the lower diagnostic yield compared with other reports.

Three cases of CMV retinitis which were newly identified were 1 eye of malignant lymphoma patient during chemotherapy, and 2 eyes of uncontrolled diabetic patient with diabetic retinopathy. Generally, HIV patients become immunocompromised host, and develop CMV retinitis.^[25]^ However, it has been known that uncontrolled diabetic patients or malignant lymphoma patients during chemotherapy result in immune dysfunction, which induces CMV retinitis.^[26,27]^ CMV retinitis in HIV patients are decreasing by highly active anti-retroviral therapy (HAART),^[28,29]^ but that uncontrolled diabetic patients and patients with hematological disease during chemotherapy would increase by aging society and progress of medical treatment.

Although prognosis of VA varied depending on the causative organism, patient condition, and timing of surgery, postoperative VA in the infectious uveitis group was significantly lower than that in the noninfectious or unidentified uveitis group. Especially, 1 eye diagnosed with CMV retinitis and 1 with acute retinal necrosis had the worst postoperative VA of less than hand motion. In addition, since PPV for cases with a preoperative presumptive diagnosis of infectious uveitis was 75%, the patients should be consulted regarding the necessity of performing diagnostic vitrectomy and the potential postoperative poor VA. However, visual acuity was improved in 9 of 13 eyes (69.2%) in the infectious uveitis group at 6 months after surgery, and postoperative VA and inflammation score improved significantly compared with the preoperative levels. These results may encourage not only the surgeons but also the patients in performing diagnostic vitrectomy.

Postoperative VA improved significantly compared with preoperative VA in the noninfectious and unidentified uveitis groups, and the VA improvement rate at 6 months after surgery was 76.2% in the noninfectious uveitis group and 90.9% in the unidentified uveitis group. In addition, there were no significant differences between preoperative and postoperative inflammation scores in both groups. These results indicate that diagnostic and therapeutic vitrectomy is effective for VO that is not controlled by topical and systemic therapies, even when the specific etiology is not infectious.

Postoperative ocular inflammation score decreased significantly compared with the preoperative score in the infectious uveitis group, but no decrease was observed in the noninfectious and unidentified uveitis groups. As shown in Fig. 5A, preoperative inflammation score in the infectious uveitis group was significantly higher than that in the noninfectious uveitis group and was apparently higher (not significantly different) than that in the unidentified uveitis group. These results suggest that vitrectomy was performed during the remission phase of ocular inflammation in the most of noninfectious and unidentified uveitis groups and during the active phase in the infectious uveitis group.

In the present study, infectious agents were identified by comprehensive PCR analysis using multiple primers for HHV1–8, bacterial 16S and fungal 18S/28S rDNA, while intraocular antibodies were not measured. As demonstrated by Sugita et al^[30]^ and Ogawa et al,^[31]^ >10 types of infectious uveitis can be identified by this procedure, and the sensitivity, specificity, positive predictive value, and negative predictive value were 91.3%, 98.8%, 98.6%, and 92.4%, respectively.^[8]^

There are possible sources of bias in this study. Since most of these patients were referred to our hospital which is specialized in uveitis and vitroretinal diseases, this referral bias may increase the likelihood of encountering patients with VO induced by ocular inflammation. The prevalence of uveitis seen in our patient population also may have affected the diagnoses in this study. In addition to the limitations of a retrospective case series, this study analyzes the case files of a single center. The decision to perform diagnostic vitrectomy, the timing of this procedure, and the choice of analyses using vitreous samples may differ among centers. Moreover, the threshold at which diagnostic and therapeutic vitrectomy is performed would be dictated not only by the clinical features of individual cases, but also by individual surgeons’ experience with the procedure. A third party that decides the tests using vitreous specimens would have minimized this bias.

In summary, for challenging cases of VO in which clinical examinations and systemic laboratory workup failed to identify the cause of the intraocular inflammation, diagnostic and therapeutic vitrectomy was useful in establishing a definitive diagnosis and improving VA outcome. Identification of the specific etiology causing the inflammation guides ophthalmologists to consider more specific local and systemic therapies, and rules out infection and malignancy allowing ophthalmologists to proceed more confidently in treating the patients with a nonspecific inflammatory condition. The usefulness of diagnostic and therapeutic vitrectomy may improve by further development of detection methods and pharmacological agents.

## Acknowledgments

The authors thank all patients who participated in this study and also Kanako Tonegawa, Tomomi Nakamura, Seiko Yamada, Saeko Kanno, and Eiko Machida for technical assistance.
